# Among the Boards of Examiners

**Published:** 1899-08

**Authors:** 


### AMONG THE BOARDS OF EXAMINERS.

### VETERINARIANS PASSING EXAMINATION OF STATE BOARDS OF VETERINARY MEDICAL EXAMINERS AND RECEIVING LICENSES SINCE JANUARY 1, 1899.

*Pennsylvania.* Liberty L. Cheney, Augusta, Ga.; Chas. S. Gelbert, Jr., Scranton, Pa.; Herbert Hoopes, Bynum, Md.; Walter G. Huyett, Wernersville, Pa.; Moses Jacob, Philadelphia, Pa.; Clinton F. Keiter, Williamstown, Pa.; John P. Miller, Lebanon, Pa.; Ezra W. Newcomer, Mt. Joy, Pa.; Henry W. Peter, Saegersville, Pa.; Jos. F. Price, Cogan Station, Pa.; D. Ryder, Chambersburg, Pa.; Frederick Taylor, Sewickley, Pa.; N. L. Townsend, Camden, Del.; G. A. Wehr, Denver, Pa.

*Maryland.* L. E. Almony, White Hall, Baltimore Co., Md.; Herbert Hoopes, Bynum, Md.; J. N. Megary, 1614 Linden Ave., Baltimore, Md.

*Ohio.* Edgar H. Callander, Zanesville, Ohio; Aubrey B. Detchon, Boardman, Ohio; Joseph King, Brice, Ohio; Wm. T. Sparhawk, Akron, Ohio; C. J. Williamson, Bucyrus, Ohio.

*North Dakota.* Duncan McKenzie, Drayton, N. D.; Herbert von Gorris, Grafton, N. D.

*New York.* P. A. Fish, Ithaca, N. Y.; Carl W. Gay, Ithaca, N. Y.; Henry W. Illston, Ithaca, N. Y.; Chester R. Perkins, Hardys, N. Y.; F. G. Scammel, Lafayette, N. Y.; William K. Ingles, Massena, N. Y.; William S. Stevenson, Tyre, N. Y.; William S. Johnson, Brooklyn, N. Y.

---

The Ohio State Board holds its regular meetings in April and July of each year. A special meeting will be held in September to examine a number of applicants.

Pennsylvania Board meets to examine applicants the third Monday and Tuesday following of June and December of each year. This affords those failing in June an opportunity of coming up in December after an expiration of six months, as provided for in the law.

The Maryland State Board holds its annual meeting in June of each year and at other times fixed by officers of the Board.

The North Dakota State Board of Veterinary Medical Examiners find an urgent field of work before them. This State has been the "Mecca" of a type of quacks and impostors that has brought the profession in great disrepute. The members of this board are: President, Dr. E. J. Davidson, Grand Forks; Secretary and Treasurer, Dr. J. N. Sheppard, Langdon, and F. H. Farmer, Wahpeton, N. D.

Illinois Board of Veterinary Examiners are: M. H. McKillip, M.D., V.S., President of the McKillip Veterinary College; James Robertson, D.V.S., President of the Chicago Veterinary Society, and C. H. Merrick, D.V.S., of Okawville, Ills. Dr. McKillip fills the röle of Chairman, and Mr. C. P. Johnson, Secretary of the Board of Livestock Commissioners, fills the same röle for the Examiners. The boards are preparing a list of those entitled to licenses, which will probably reach 600. It is believed by those who have pinned their faith to this law that its association with the Livestock Commissioners will prove a source of great strength, in that it will be provided with funds for the carrying out of the provisions of the act.

